# Spectroscopic Properties of Inorganic Glasses Doped with Pr^3+^: A Comparative Study

**DOI:** 10.3390/ma15030767

**Published:** 2022-01-20

**Authors:** Joanna Pisarska, Marta Kuwik, Wojciech A. Pisarski

**Affiliations:** Institute of Chemistry, University of Silesia, Szkolna 9 Street, 40-007 Katowice, Poland; marta.kuwik@us.edu.pl (M.K.); wojciech.pisarski@us.edu.pl (W.A.P.)

**Keywords:** inorganic glasses, Pr^3+^ ions, luminescence, spectroscopic properties

## Abstract

The results presented in this communication concern visible and near-IR emission of Pr^3+^ ions in selected inorganic glasses, i.e., borate-based glass with Ga_2_O_3_ and BaO, lead-phosphate glass with Ga_2_O_3_, gallo-germanate glass modified by BaO/BaF_2_, and multicomponent fluoride glass based on InF_3_. Glasses present several emission bands at blue, reddish orange, and near-infrared spectral ranges, which correspond to 4f–4f electronic transitions of Pr^3+^. The profiles of emission bands and their relative intensity ratios depend strongly on glass-host. Visible emission of Pr^3+^ ions is tuned from red/orange for borate-based glass to nearly white light for multicomponent fluoride glass based on InF_3_. The positions and spectral linewidths for near-infrared luminescence bands at the optical telecommunication window corresponding to the ^1^G_4_ → ^3^H_5_, ^1^D_2_ → ^1^G_4_, and ^3^H_4_ → ^3^F_3_,^3^F_4_ transitions of Pr^3+^ are dependent on glass-host matrices and excitation wavelengths. Low-phonon fluoride glasses based on InF_3_ and gallo-germanate glasses with BaO/BaF_2_ are excellent candidates for broadband near-infrared optical amplifiers. Spectroscopic properties of Pr^3+^-doped glasses are compared and discussed in relation to potential optical applications.

## 1. Introduction

Praseodymium-doped inorganic glasses, due to several visible and near-infrared emission transitions, are interesting from the spectroscopic point of view. Systematic studies demonstrate that radiative and non-radiative relaxation from the excited states of Pr^3+^ ions depend significantly on the glass-host matrices. These aspects for borate [1], phosphate [2], silicate [3], tellurite [4], germanate [5], and other non-oxide glass systems [6,7,8,9,10] are well documented in literature. Emission properties of Pr^3+^-doped glasses have been examined at visible wavelengths [11,12,13,14,15,16,17] and the near-infrared (NIR) region [18,19,20,21]. Most published papers are related to luminescence spectroscopy of Pr^3+^ ions in glasses belonging to the heavy metal glass family [22,23,24,25,26,27,28]. Special attention has been paid to Pr^3+^ ions in silicate glass containing lead [29,30,31]. Further comprehensive investigations indicate that the emission bands associated with electronic transitions of Pr^3+^ ions are enhanced in the presence of silver [32,33,34,35] or gold [36] nanoparticles embedded into glass matrices.

Here we present comparative studies on selected inorganic glasses containing Pr^3+^, i.e., borate glass with Ga_2_O_3_ and BaO, lead-phosphate glass with Ga_2_O_3_, gallo-germanate glass modified by BaO/BaF_2_, and multicomponent fluoride glass based on InF_3_. Based on luminescence spectra and their decays, several spectroscopic parameters of Pr^3+^ ions were determined. Previous investigations illustrated quite well the relationship between the structural modifications of glasses and their emission and spectroscopic properties. For example, several glass-modifiers were introduced to borate glasses doped with Pr^3+^ ions. Anjaiah et al. [37] studied luminescence properties of Pr^3+^-doped lithium borate glasses modified by MO (where M = Zn, Ca, Cd). Based on some spectroscopic parameters such as the Judd–Ofelt intensity parameter Ω_2_ and the bonding parameter δ, it was suggested that the covalent environment for Pr^3^^+^ increased in the following direction CdO < CaO < ZnO, and glass modified by CdO becomes a better candidate for thermoluminescence among the three studied Pr^3^^+^-doped glass systems. Spectroscopic properties of Pr^3+^ are also changed during modification of the borate glass composition with lithium oxide and fluoride. Jayasankar and Babu [38] revealed that the radiative lifetimes for the excited states of Pr^3+^ ions are reduced with decreasing lithium oxide concentration, while their values increase with increasing LiF content. The local structure and some properties of borate glasses are also changed with Li_2_O [39] and Na_2_O [40], respectively.

Furthermore, Pr^3+^-doped borate-based glasses modified by MO (M = Ca, Sr, Ba) have also been studied using emission spectroscopy. The emission bands related to the ^1^D_2_ → ^3^H_4_ transition of Pr^3^^+^ ions are slightly shifted to lower wavelengths (nephelauxetic effect), and the ^1^D_2_ measured lifetimes are reduced in the presence of glass-modifiers in the direction BaO → SrO → CaO [41]. Further modification of borate glass realized by replacement of BaO by BaF_2_ results in spectral shift of the reddish orange ^1^D_2_ → ^3^H_4_ transition of Pr^3+^ ions toward shorter wavelengths [42]. The changes in luminescence decays, profiles of emission bands, and their relative intensity ratios will be stronger for glass-host matrices, including different glass-formers. These effects were examined previously for some glass systems singly doped with Tm^3+^ [43], Sm^3+^ [44], Dy^3+^ [45], Yb^3+^ [46], and glass co-doped with Yb^3+^/Er^3+^ [47]. The intention of our work is to present how kinds of glass-host matrix influence the spectral profiles of luminescence bands of Pr^3+^ ions and their relative intensity ratios measured in the visible and near-infrared ranges. Based on spectroscopic parameters of Pr^3+^ ions, the glass-host matrices are selected as promising materials for multicolor visible light sources or broadband near-infrared optical amplifiers. 

## 2. Materials and Methods

Selected inorganic glasses doped with Pr^3+^ ions were synthesized using traditional high-temperature melt-quenching technique. Their chemical compositions and melting conditions are given in Table 1. For the studied glass samples, the activator concentration was the same (0.1 mol%). Pr^3+^-doped lead-phosphate glass with Ga_2_O_3_ (PPG-Pr) was also selected to study reddish orange emission varying with activator concentration. Samples of PPG-Pr with various Pr^3+^ concentrations were prepared. They are given in Table 2. The appropriate precursor metal oxides and/or fluorides of high purity (99.99%) were mixed in a Pt crucible and then melted in a special glove-box in an Ar atmosphere. Glass samples with dimension = 10 mm × 10 mm and thickness = 2 mm were obtained.

In the next step, absorption and luminescence measurements were carried out. The UV-VIS-NIR spectrophotometer (Cary 5000, Agilent Technology, Santa Clara, CA, USA) was used to measure absorption spectra. Luminescence spectra and their decays were registered using a VIS/NIR laser system. The laser equipment consisted of a Photon Technology International (PTI) Quanta-Master 40 (QM40) UV/VIS Steady State Spectrofluorometer (Photon Technology International, Birmingham, NJ, USA) coupled with tunable pulsed optical parametric oscillator (OPO), pumped by a third harmonic of a Nd:YAG laser (Opotek Opolette 355 LD, OPOTEK, Carlsband, CA, USA), xenon lamp as a light source, double 200 mm monochromator, multimode UVVIS PMT R928 detector (PTI Model 914), and Hamamatsu H10330B-75 detector (Hamamatsu, Bridgewater, NJ, USA). Resolution for spectra measurements was ±0.2 nm. Decays were measured with an accuracy of ±2 µs. Transmittance spectra were performed on the Nicolet iS50 ATR spectrometer (Thermo Fisher Scientific Instruments, Waltham, MA, USA). 

## 3. Results and Discussion

**Figure 1:**
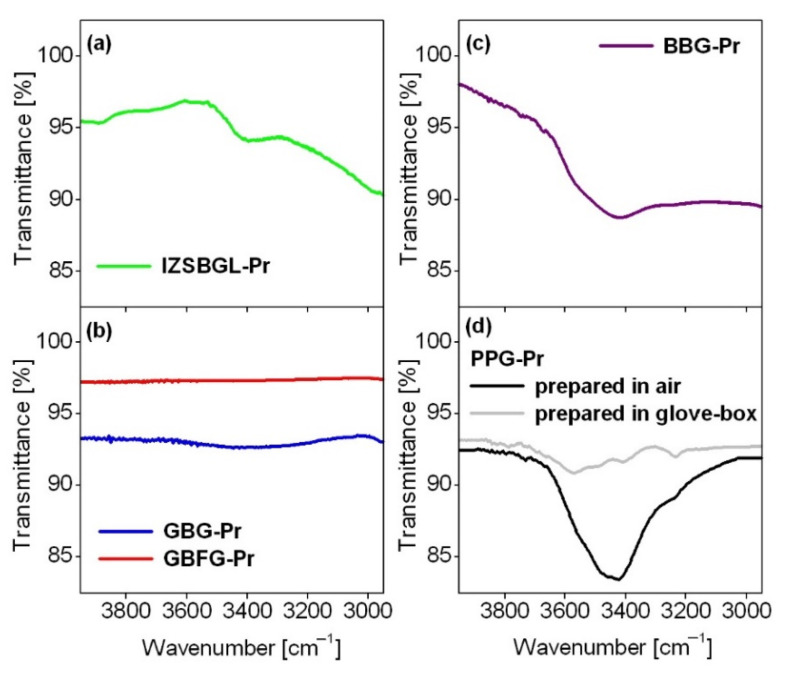
Transmittance spectra measured for the studied glass samples. IZSBGL-Pr (**a**), GBG-Pr and GBFG-Pr (**b**), BBG-Pr (**c**) and PPG-Pr (**d**).

Five glass-host matrices given in Table 1 and referred to as IZSBGL-Pr, GBFG-Pr, GBG-Pr, PPG-Pr, and BBG-Pr were selected for comparative spectroscopic investigations. It should be noted that all glass samples were obtained under the same experimental conditions in order to compare their spectroscopic properties. It is well known that the conditions of synthesis are more restrictive for pure fluoride glasses in contrast to oxide glass systems. In our case, all samples were prepared in a glove-box under an atmosphere of dry argon (O_2_, H_2_O < 0.5 ppm). This procedure is especially important for IZSBGL-Pr, due to fluorine evaporation during the glass synthesis. For that reason, a small amount of ammonium bifluoride (NH_4_HF_2_) as a fluorinating agent was also added before melting. Unfortunately, the actual concentration of fluorine ions has not been estimated. The final composition of IZSBGL-Pr may be somewhat different from the nominal starting one due to fluorine losses during the melting process. Previously published works suggest that the fluorine losses could be quite large [48,49,50,51,52]. An another important factor that effectively quenched the luminescence is the concentration of OH^-^ groups, which can be calculated from the transmittance spectrum. Figure 1 shows transmittance spectra measured for glass samples in the 3950–2950 cm^−1^ frequency region. The absorption band centered at about 3400 cm^−1^ is ascribed to the vibration of OH^-^ groups.

For fluoride glass based on InF_3_, the concentration of OH^–^ groups is extremely low. The absorption coefficient and content of hydroxyl groups are close to 0.088 cm^−1^ and 3.82 ppm [53], respectively. The reduced concentration of hydroxyl groups is necessary to obtain pure fluoride glass with relatively high quantum efficiency and to enhance near-IR and mid-IR emission [54]. Further investigations indicate that the band intensity of hydroxyl groups is considerably smaller for mixed oxyfluoride gallo-germanate glass with BaF_2_ (GBFG-Pr) than oxide glass (GBG-Pr). The residual absorption of OH^-^ groups is reduced drastically in gallo-germanate glass, where BaO was replaced by BaF_2_ [55]. These aspects are also important for phosphate glasses due to the hygroscopic nature of P_2_O_5_. The concentration of hydroxyl groups is usually higher in phosphate glass compared to other oxide glasses. Our recent studies for the lead-phosphate system [56] clearly demonstrated that the intensity of the IR band related to vibration of hydroxyl groups is considerably lower for glass samples synthesized in glove-box than in open air. These phenomena are very important from the optical point of view. Based on our published works [56,57,58,59], different physicochemical properties of the studied Pr^3+^-doped glasses are also summarized in Table 3.

**Figure 2:**
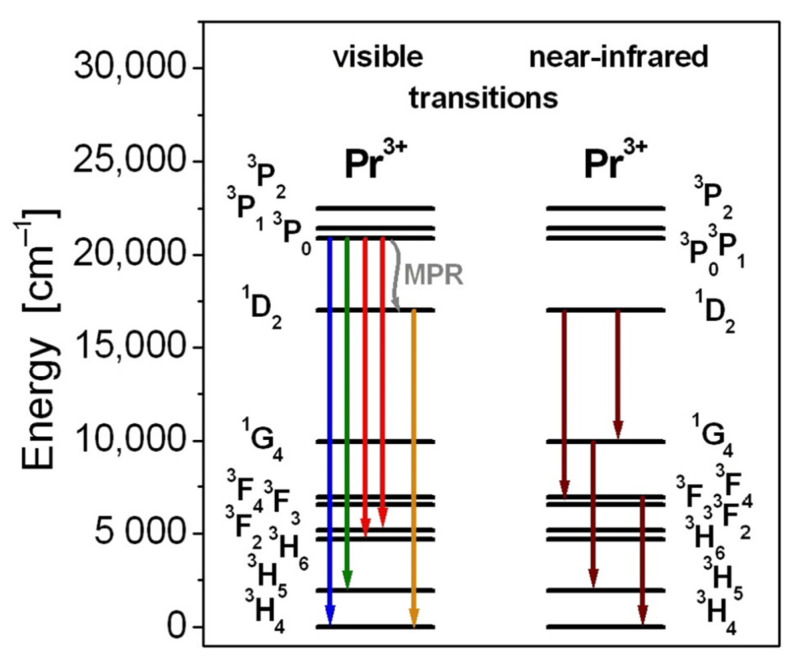
Energy level diagram of praseodymium ions in inorganic glasses.

From the average molecular weight, density, Pr^3+^ ion concentration, and refractive index exhibited in Table 3, various other radiative parameters were calculated. The three phenomenological intensity parameters Ω_t_ (where t = 2, 4, 6) were calculated by using the appropriate relations from the Judd–Ofelt (J–O) theory. In particular, the J–O intensity parameter Ω_2_ is attributed to the sensitivity to the local glass structure of the rare earth sites. It is affected by symmetry/asymmetry sites and covalent/ionic bonding character between Pr^3+^ ions and the nearest surroundings. In other words, the lower values of Ω_2_ suggest a higher degree of ionic bonding between rare earth ions and their ligands. It is clearly seen that the value of Ω_2_ is greater for glass GBG-Pr, in contrast to fluoride glass IZSBGL-Pr and oxide glasses assigned to PPG-Pr and BBG-Pr, suggesting a higher degree of covalence between Pr^3+^ ions and the surrounding ligands. Independently of glass-host, the radiative transition rates obtained from the J–O calculations are significantly higher for the ^3^P_0_ state than the lower-lying ^1^D_2_ state of Pr^3+^ ions. Further calculations from the relevant expression η = τ_m_/τ_rad_ × 100% (τ_m_ and τ_rad_ are measured and radiative lifetime, respectively, calculated from the J–O theory) indicate that the quantum efficiency for the excited state ^1^D_2_ (Pr^3+^) is significantly larger for low-phonon oxide (GBG-Pr) and fluoride (IZSBGL-Pr) glasses, confirming their suitability for near-infrared luminescence applications. Glass transition temperature T_g_ for the studied glass-host matrices was also determined from DSC curve measurements. The value of T_g_ is much lower for fluoride glass IZSBGL-Pr compared to other systems. Glass GBG-Pr is characterized by the highest glass transition temperature among the studied glass systems. It is also interesting to note that the value of T_g_ changes from 620 °C (GBG-Pr) to 599 °C (GBFG-Pr) in gallo-germanate glass where BaO was partially substituted by BaF_2_ [59]. Furthermore, the energy level diagram for Pr^3+^ ions schematized in Figure 2 favors several visible and near-infrared emission transitions.

The spectroscopic results for Pr^3+^ ions in fluoride glass based on InF_3_ (IZSBGL-Pr), borate glass with Ga_2_O_3_ and BaO (BBG-Pr), lead-phosphate glass with Ga_2_O_3_ (PPG-Pr), and gallo-germanate glassses modified by BaO/BaF_2_ (referred to as GBG-Pr and GBFG-Pr) are presented and discussed here.

**Figure 3:**
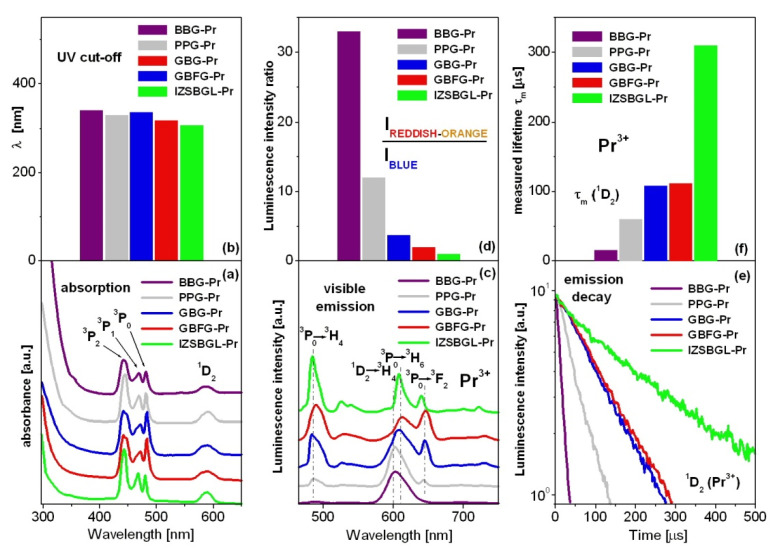
Absorption (**a**,**b**) and visible emission (**c**,**d**) spectra and emission decay curves (**e**,**f**) for Pr^3+^ ions in inorganic glasses.

Figure 3 presents absorption (a,b) and visible emission (c,d) spectra and emission decays (e,f) from ^1^D_2_ state of Pr^3+^ ions in the studied glass systems. Absorption spectra consist of characteristic bands which correspond to transitions originating from ground state ^3^H_4_ to the higher-lying excited states of praseodymium ions. The most intense bands centered at 445 nm and 590 nm are related to ^3^H_4_ → ^3^P_2_ and ^3^H_4_ → ^1^D_2_ transitions of Pr^3+^, respectively. The UV cut-off wavelength, defined as the intersection between the zero baseline and the extrapolation of absorption edge, is located in the 300–350 nm range. In general, the absorption edge is shifted to shorter wavelengths from oxide borate glass BBG-Pr to fluoride glass IZSBGL-Pr. Visible emission spectra were excited at ^3^P_2_ state (λ_exc_ = 445 nm) and show several characteristic bands of Pr^3+^ ions. The most intense bands are located in the blue and reddish orange spectral ranges and correspond to ^3^P_0_ → ^3^H_4_, ^1^D_2_ → ^3^H_4_, ^3^P_0_ → ^3^H_6_, and ^3^P_0_ → ^3^F_2_ electronic transitions of Pr^3+^.

Further analysis demonstrates that the relative integrated intensities of emission bands located in the blue and reddish orange region are completely different and depend strongly on kind of glass-host. Previous studies revealed that fluorescence intensity ratio, referred to as red-to-blue R/B [60] or orange-to-blue O/B [61], decreases with increasing Pr^3+^ ion concentration. In our case, fluorescence intensity ratio I_REDDISH-ORANGE_/I_BLUE_ varying with glass-host was also estimated and schematized in Figure 3. This factor is enhanced rapidly from fluoride glass IZSBGL-Pr to borate-based glass BBG-Pr, due to the increase in the non-radiative rates. As a consequence, the ^3^P_0_ state is depopulated very quickly, and the excitation energy is transferred non-radiatively to the lower-lying state ^1^D_2_ (Pr^3+^). It can be well explained by the phonon energy of the host (Table 3), which increases from 510 cm^−1^ (IZSBGL-Pr) to 1400 cm^−1^ (BBG-Pr). Thus, high-phonon borate glass BBG-Pr is favored to bridge the energy gap between ^3^P_0_ and ^1^D_2_ states of Pr^3+^ ions, and reddish orange emission due to ^1^D_2_ → ^3^H_4_ transition is dominant. This was also confirmed by luminescence decay analysis. The multi-phonon relaxation rates of Pr^3+^ increase with increasing phonon energy from IZSBGL-Pr to BBG-Pr. Owing to higher multi-phonon relaxation rates, the measured luminescence lifetimes of ^1^D_2_ (Pr^3+^) are reduced from fluoride glass IZSBGL-Pr to borate-based glass BBG-Pr. Furthermore, luminescence decays from ^1^D_2_ state in all glass samples containing 0.1 mol% Pr^3+^ ions are mono-exponential. According to the excellent review article published recently by Tanner et al. [62], mono-exponential decay using the Förster expression for W_ET_ can be given for electric-dipole type transfer by:

(1) $$I_{D}\left(t\right)=I_{D}\left(0\right)\exp\left[-\left(\frac{1}{\tau_{D}}+W_{\mathrm{ET}}\right)t\right]\;$$

or the following relation:

(2) $$I_{D}\left(t\right)=I_{D}\left(0\right)\exp\left[\left(-1-{\left(\frac{R_{0}}{R}\right)}^{6}\right)\left(\frac{t}{\tau_{D}}\right)\right]$$

where R_0_ is critical transfer distance (also called Förster radius), R is the average interionic separation, equal to (3/4πN)^1/3^, and N denotes activator concentration. 

The energy transfer and cross-relaxation processes are neglected when the average interionic separation R between Pr^3+^ ions is greater than the critical transfer distance R_0_. Our studies clearly indicate that calculated values of R for all studied glass-host matrices containing 0.1 mol% Pr^3+^ ions are greater than the Förster distances R_0_ (Table 3). Previous results obtained for Pr^3+^-doped ZBLAN fluoride glass suggest that the average distance is smaller than the critical transfer distance and the energy transfer process will promote the non-exponential decay from the ^1^D_2_ state for activator (Pr^3+^) content ≥ 0.5 mol% [63]. 

**Figure 4:**
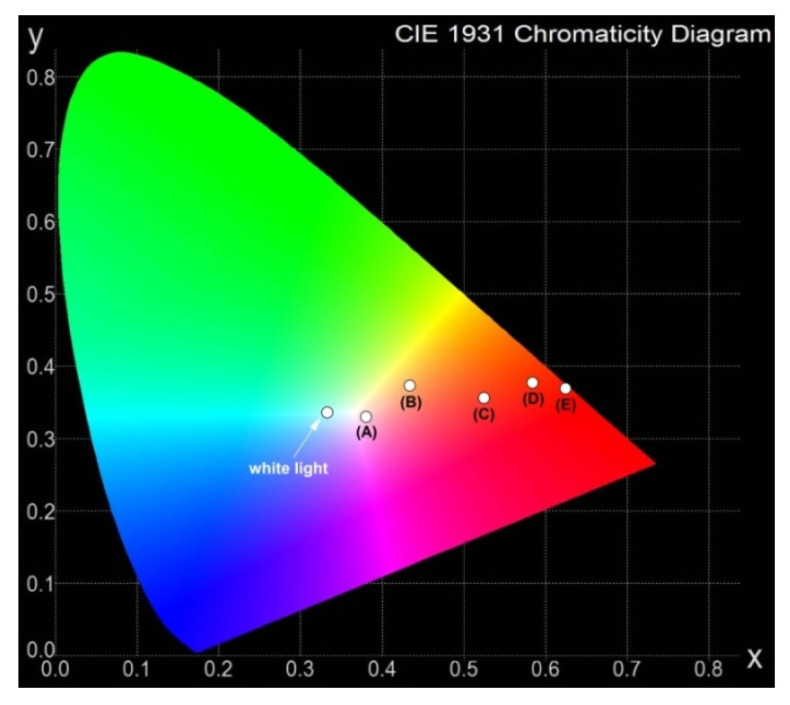
CIE chromaticity coordinates for IZSBGL-Pr (**A**), BGFG-Pr (**B**), BGG-Pr (**C**), PPG-Pr (**D**), and BBG-Pr (**E**) glass systems.

Among inorganic glass systems, it is also found that the measured ^1^D_2_ luminescence lifetime is longer than the ^3^P_0_ lifetime of Pr^3+^ ions. This was confirmed by luminescence decay curve measurements for Pr^3+^ ions in multicomponent fluoro-phosphate glasses [15], oxyfluoroborate glasses [17], lead germanate glasses [23], and borosilicate glasses [61] as well as tellurite [64] and zinc telluro-fluoroborate [65] glass systems. Luminescence lifetimes for ^3^P_0_ and ^1^D_2_ states of Pr^3+^ ions in different glass-host matrices are presented in Table 4. Also, the x and y of CIE chromaticity coordinates for IZSBGL-Pr, GBFG-Pr, GBG-Pr, PPG-Pr, and BBG-Pr systems were calculated from the emission spectra. The results are given in Table 5. They are shown in the chromaticity diagram in Figure 4.

Spectroscopic studies indicate that PPG-Pr and BBG-Pr belong to inorganic glasses emitting reddish orange emission, similar to other lead-free and lead-based [66,67,68,69] glass systems doped with Pr^3+^ published recently. It is noteworthy that the color of emission is changed from reddish orange (BGB-Pr) to yellowish orange (BGFG-Pr) where BaO was replaced by BaF_2_ (5 mol%). Based on the CIE diagram, we can conclude that emission can be tuned from red/orange (BBG-Pr) to nearly white light region (IZSBGL-Pr) by changing chromaticity parameters by varying the glass-host matrix.

**Figure 5:**
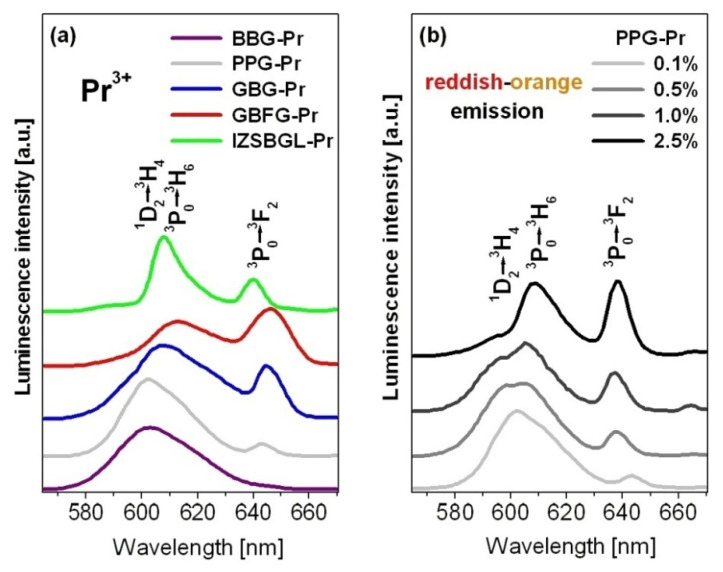
Reddish orange emission as a function of glass-host (**a**) and Pr^3+^ concentration (**b**).

Our previous investigations suggested that the spectral profiles of emission bands of Pr^3+^ ions and their relative intensity ratios are changed during modification of glass-host. In the orange-red region, two emission bands due to ^1^D_2_ → ^3^H_4_ (orange) and ^3^P_0_ → ^3^H_6_ (red) transitions of Pr^3+^ are overlapped, and their intensities depend strongly on the glass-host. This was well evidenced for gallo-germanate glasses modified by BaO/BaF_2_ [70]. Figure 5 shows reddish orange emission spectra dependent on glass-host matrix and Pr^3+^ content.

From literature data, it is well known that multi-phonon relaxation (MPR) and cross relaxation (CR) processes play an important role in population or depopulation of the ^1^D_2_ state of Pr^3+^ ions in inorganic glasses. The non-radiative transition rate W_nr_ due to the MPR process is equal to 2.47 × 10^4^ s^−^^1^ for glass based on InF_3_ [71], whereas the value of W_nr_ for borate glass is approximately 10^3^ times larger than that of the fluoride glass system [72]. The phonon energy of the host increases from IZSBGL-Pr to BBG-Pr. Thus, the excitation energy is transferred more efficiently from the higher-lying ^3^P_0_ state to the ^1^D_2_ state, and, consequently, reddish orange luminescence corresponding to ^1^D_2_ → ^3^H_4_ transition of Pr^3+^ in borate-based glass (BBG-Pr) is dominant, as mentioned above. This situation is observed for glasses when the molar concentration of Pr^3+^ ions is relatively low and its value is close to 0.1 mol%. It is generally accepted that the MPR process from the ^3^P_0_ state at lower concentrations (usually below 0.5 mol%) favors reddish orange emission from the ^1^D_2_ state to be more dominant [61]. For higher activator concentrations (above 0.5 mol%), the non-radiative energy transfer processes between Pr^3+^ ions become efficient, and luminescence associated to ^1^D_2_ → ^3^H_4_ transition is successfully quenched through cross-relaxation. The following CR processes, ^1^D_2_: ^3^H_4_ → ^1^G_4_: (^3^F_3_,^3^F_4_) and ^1^D_2_: ^3^H_4_ → (^3^F_3_,^3^F_4_): ^1^G_4_, are responsible for depopulation of the ^1^D_2_ state of Pr^3+^ [73]. In addition, these aspects have been examined by us. In our case, lead-phosphate glass (PPG-Pr) was selected as an intermediate medium, in which luminescence from both ^3^P_0_ and ^1^D_2_ states of Pr^3+^ ions are well observed and the ^1^D_2_ → ^3^H_4_ transition is dominant at low activator concentration. The results are presented in Figure 5b. It is well evidenced that the emission intensity of ^1^D_2_ → ^3^H_4_ transition is reduced, whereas the emission intensities of bands originating from the ^3^P_0_ state are enhanced with increasing Pr^3+^ concentration. These phenomena are associated with Pr^3+^–Pr^3+^ interaction increasing and the presence of cross-relaxation processes at higher activator concentration. A similar situation was observed for zinc-telluro-fluoroborate glass examined as a function of Pr^3+^ ion concentration [66]. Our experimental results evidently suggest that the contribution of the glass-host to the change in the spectral factor I_REDDISH-ORANGE_/I_BLUE_ and measured lifetime (Figure 3) seems to be dominant when content of Pr^3+^ is relatively low (0.1 mol%). In this case, the multi-phonon relaxation process makes an important contribution to the excited state relaxation of Pr^3+^ (Figure 5a). The situation was completely changed when concentration of rare earths was relatively high (Figure 5b). Thus, the contribution of activator content was dominant. This behavior is due to the presence of non-radiative energy transfer processes (such as cross-relaxation), which contribute to quenching of luminescence corresponding to ^1^D_2_ → ^3^H_4_ transition of Pr^3+^.

**Figure 6:**
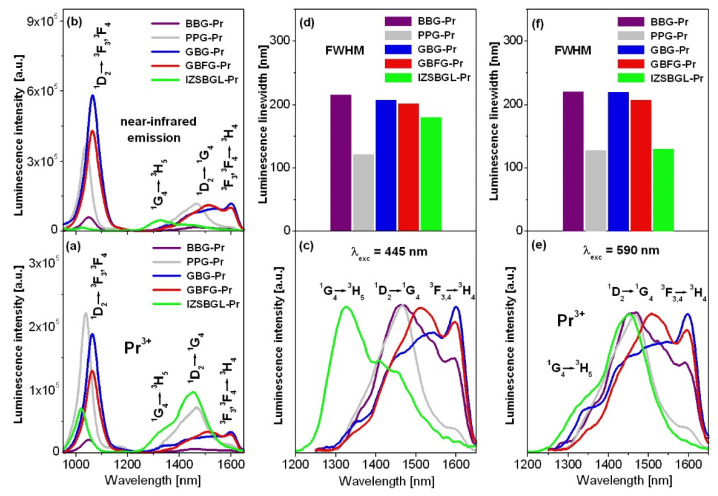
Near-infrared luminescence spectra of Pr^3+^ ions in inorganic glasses (**a**,**b**). Normalized spectra in 1200–1650 nm range excited at 445 nm (**c**,**d**) and 590 nm (**e**,**f**) are also indicated.

Figure 6 presents near-infrared emission spectra of Pr^3+^ ions in inorganic glasses, which were excited at 445 nm (^3^P_0_) and 590 nm (^1^D_2_), respectively. In order to compare luminescence linewidth, defined as full width at half maximum (FWHM), the spectra measured in the 1200–1650 nm range were also normalized.

The near-infrared luminescence spectra show several bands which correspond to ^1^D_2_ → ^3^F_3_,^3^F_4_, ^1^G_4_ → ^3^H_5_, ^1^D_2_ → ^1^G_4_, and ^3^H_4_ → ^3^F_3_,^3^F_4_ transitions of Pr^3+^, respectively. Their relative integrated emission intensities are changed drastically with glass-host matrices. In particular, luminescence bands located in the so-called telecom window (1200–1650 nm) have been examined in detail. In this spectral range, ultra-broadband near-infrared emission of Pr^3+^ ions related to ^1^G_4_ → ^3^H_5_ (λ_p_ = 1330 nm) and ^1^D_2_ → ^1^G_4_ (λ_p_ = 1480 nm) transitions is observed for several inorganic glasses, which is extremely useful for optical fiber amplifiers operating at E-, S-, C-, and L-band [74]. In some cases, a near-infrared emission band centered at about 1600 nm is also visible. This emission band is connected with the ^3^H_4_ → ^3^F_3_,^3^F_4_ transition of Pr^3+^ [75]. Interesting results are observed for fluoride glass IZSBGL-Pr. In contrast to other studied glass systems, the intensities of emission bands of Pr^3+^ ions in glass IZSBGL-Pr, covering a spectral range from 1200 nm to 1650 nm, depend also on the excitation wavelengths (445 nm/590 nm). When glass IZSBGL-Pr was excited at 445 nm (^3^P_2_), the intensities of bands were extremely low, and the near-infrared emission near 1335 nm due to the ^1^G_4_ → ^3^H_5_ transition was dominant. The situation changed when the glass sample was excited at 590 nm (^1^D_2_). Thus, the near-infrared emission in glass IZSBGL-Pr is the most intense, and the ^1^D_2_ → ^1^G_4_ transition centered at about 1450 nm is dominant.

Independently of excitation wavelengths, broadband near-infrared emission bands (FWHM above 200 nm) are observed for BBG-Pr, GBG, and GBFG glasses. From the literature, it is well known that near-infrared luminescence properties of glasses containing Pr^3+^ ions depend strongly on the excitation wavelengths. The blue and orange excitation lines are unusually helpful to examine conversion of blue light into near-infrared radiation and its mechanism. These processes have been observed for some fluoride materials and other low-phonon systems. The experimental results for glass-ceramic materials with CaF_2_:Pr^3+^ nanocrystals [76] indicated that a two-step near-infrared quantum cutting (NIR-QC) from blue-excited ^3^P_0_ state takes place efficiently, with ^1^G_4_ acting as an intermediate state. Blue light excitation leading to efficient population of ^1^G_4_ state also influences the relative integrated intensities of emission bands, which correspond to near-infrared transitions originating from both ^1^D_2_ and ^1^G_4_ states of Pr^3+^. A tunable amplification band depending on the excitation wavelength used (474 nm/980 nm) has been also observed for Pr^3+^/Yb^3+^ co-doped systems, where it is possible to select ^1^D_2_ → ^1^G_4_ or ^1^G_4_ → ^3^H_5_ transition of Pr^3+^. When the excitation wavelength was changed from 474 nm to 980 nm, the near-infrared luminescence switched from the E–S bands near 1480 nm to the O–E bands centered at 1330 nm in Pr^3+^/Yb^3+^ co-doped tellurite tungstate glasses [77].

Finally, some spectroscopic parameters for Pr^3+^ ions were determined. One of the most important radiative parameters is the peak stimulated emission cross-section σ_em_, which can be calculated using the expression:

(3) $$\sigma_{\mathrm{em}}=\frac{\lambda_{p}^{4}}{8{\pi \mathrm{cn}}^{2}\Delta \lambda }\;A_{J}$$

where λ_p_ is the peak emission wavelength, n—the refractive index, c—the velocity of light, Δλ—the emission linewidth (FWHM), and A_J_—the calculated radiative transition rate from the J–O theory. The values of n and A_J_ are given in Table 3. It is generally accepted that a relatively large value of σ_em_ is demanded for an efficient laser transition.

In the next step, the stimulated emission cross-section (σ_em_), the measured emission lifetime (τ_m_), and the emission linewidth (FWHM) were applied to calculate the following parameters: figure of merit FOM (σ_em_ × τ_m_) and gain bandwidth (σ_em_ × FWHM product). The results for the ^1^D_2_ → ^1^G_4_ transition of Pr^3+^ ions in the glass systems excited at 590 nm are given in Table 6.

The peak stimulated emission cross-section for PPG-Pr close to σ_em_ = 1.28 × 10^−20^ cm^2^ is relatively large and comparable to the values 1.14 × 10^−20^ cm^2^ [78] and 1.29 × 10^−20^ cm^2^ [79] reported previously for similar phosphate-based glasses doped with Pr^3+^. The smaller values of the stimulated emission cross-section (σ_em_ = 0.5 × 10^−20^ cm^2^) as well as the gain bandwidth (σ_em_ × FWHM = 65 × 10^−27^ cm^3^) for fluoride glass IZSBGL-Pr are mainly due to the considerably lower spectral linewidth for ^1^D_2_ → ^1^G_4_ transition of Pr^3+^. On the other hand, the figure of merit (FOM) for IZSBGL-Pr is the highest among the studied glass systems.

The peak stimulated emission cross-section, the figure of merit (FOM), and the gain bandwidth seem to be considerably smaller for glass BBG-Pr, due to its relatively large non-radiative transition rate. For that reason, high-phonon borate-based glass BBG-Pr is useless for near-infrared luminescence applications. The σ_em_ × FWHM product, as an important parameter to achieve broadband and high gain amplification, is quite large for GBG and GBFG glasses (above 200 × 10^−^^27^ cm^3^). Their calculated values are comparable to the one (174.6 × 10^−^^27^ cm^3^) obtained for the ^1^D_2_ → ^1^G_4_ transition of Pr^3+^ ions in fluorotellurite glass [80], demonstrating suitability for broadband near-infrared amplifiers. 

## 4. Conclusions

In this work, comparative spectroscopic properties of selected inorganic glasses singly doped with Pr^3+^ ions are reported. The experimental results were limited to borate-based glass with Ga_2_O_3_ and BaO, lead-phosphate glass with Ga_2_O_3_, gallo-germanate glass modified by BaO/BaF_2_, and multicomponent fluoride glass based on InF_3_. Spectroscopic parameters for Pr^3+^ ions in glass samples were determined based on absorption/emission spectra measurements and emission decay curve analysis. Emission spectra at visible and near-infrared wavelengths were analyzed based on the energy level diagram of Pr^3+^. The systematic studies revealed that profiles of emission bands and their relative integrated intensity ratios depend significantly on glass-host matrices. Visible emission of Pr^3+^ is modulated from red/orange for borate-based glass and lead-phosphate glass with Ga_2_O_3_ via yellowish orange for gallo-germanate glass with BaO/BaF_2_ to nearly white light for fluoride glass based on InF_3_. The band positions and spectral linewidths for near-infrared luminescence at telecom range associated with the ^1^G_4_ → ^3^H_5_, ^1^D_2_ → ^1^G_4_, and ^3^H_4_ → ^3^F_3_,^3^F_4_ transitions of Pr^3+^ are influenced by the kind of glass matrix and excitation wavelengths. Based on several spectroscopic parameters of Pr^3+^ ions, it was suggested that low-phonon fluoride glasses based on InF_3_ and gallo-germanate glasses with BaO/BaF_2_ are promising materials for optical amplification. The results are compared and discussed in relation to potential applications as multicolor visible light sources or broadband near-infrared optical amplifiers.

## Figures and Tables

**Table 1:** Chemical compositions and melting conditions for inorganic glasses doped with Pr^3+^.

Glass Code	Chemical Composition [mol%]	Melting Conditions
IZSBGL-Pr	37.9InF_3_-20ZnF_2_-20SrF_2_-16BaF_2_-4GaF_3_-2LaF_3_-0.1PrF_3_	900 °C/60 min
GBFG-Pr	60GeO_2_-25BaO-5BaF_2_-9.9Ga_2_O_3_-0.1Pr_2_O_3_	1200 °C/45 min
GBG-Pr	60GeO_2_-30BaO-9.9Ga_2_O_3_-0.1Pr_2_O_3_	1200 °C/45 min
PPG-Pr	45PbO-45P_2_O_5_-9.9Ga_2_O_3_-0.1Pr_2_O_3_	1100 °C/30 min
BBG-Pr	60B_2_O_3_-30BaO-9.9Ga_2_O_3_-0.1Pr_2_O_3_	1250 °C/45 min

**Table 2:** Chemical compositions and melting conditions for lead-phosphate glass doped with Pr^3+^.

Glass Code	Chemical Composition [mol%]	Melting Conditions
PPG-0.1Pr	45PbO-45P_2_O_5_-9.9Ga_2_O_3_-0.1Pr_2_O_3_ 45PbO-45P_2_O_5_-9.5Ga_2_O_3_-0.5Pr_2_O_3_ 45PbO-45P_2_O_5_-9.0Ga_2_O_3_-1.0Pr_2_O_3_ 45PbO-45P_2_O_5_-7.5Ga_2_O_3_-2.5Pr_2_O_3_	1100 °C/30 min
PPG-0.5Pr		1100 °C/30 min
PPG-1.0Pr		1100 °C/30 min
PPG-2.5Pr		1100 °C/30 min

**Table 3:** Different physicochemical properties of the studied Pr^3+^-doped inorganic glasses [56,57,58,59].

Parameters	Glass-Host			
	IZSBGL-Pr	GBG-Pr	PPG-Pr	BBG-Pr
Average molecular weight (M g mol^−1^)	140.94	127.63	183.22	106.63
Density (d g cm^−3^)	4.38	4.58	4.11	3.19
Pr^3+^ content (molar %)	0.1	0.1	0.1	0.1
Pr^3+^ concentration (Nx10^19^ ions cm^−3^)	1.87	4.31	2.70	3.59
Average interionic separation (R Å)	18.5	14.0	16.3	14.9
Critical transfer distance (R_0_ Å)	11.3	6.5	8.5	7.8
Refractive index (n)	1.48	1.73	1.75	1.61
Glass transition temperature (T_g_ °C)	295	620	437	566
Phonon energy of the host (hω cm^−1^)	510	790	1120	1400
Judd–Ofelt parameters Ω_t_ (10^−20^ cm^2^)				
Ω_2_	2.01	6.93	1.81	2.17
Ω_4_	5.25	19.68	18.33	9.75
Ω_6_	5.10	8.95	15.51	2.62
Radiative transition rate (A_J_ s^−1^)				
from ^3^P_0_ state (Pr^3+^)	30,200	123,050	95,250	60,450
from ^1^D_2_ state (Pr^3+^)	2440	8930	8330	3370
Quantum efficiency ^1^D_2_ Pr^3+^ (η %)	88	98	50	5

**Table 4:** Luminescence lifetimes for ^3^P_0_ and ^1^D_2_ states of Pr^3+^ ions in inorganic glasses.

Glass-Host Composition [mol%]	^3^P_0_ [µs]	^1^D_2_ [µs]	Ref.
57ZrF_4_-34BaF_2_-4AlF_3_-4.5LaF_3_-0.5PrF_3_	37	-	[8]
50SiO_2_-10Al_2_O_3_-2MgO-20CaO-15SrO-3BaO-0.1Pr_2_O_3_	115	-	[11]
74.8TeO_2_-15Sb_2_O_3_-10WO_3_-0.2Pr_6_O_11_	11.73	-	[12]
60P_2_O_5_-4B_2_O_3_-7Al_2_O_3_-10K_2_O-17.95BaO-0.05Pr_2_O_3_	-	173	[13]
49.5P_2_O_5_-10AlF_3_-10BaF_2_-10SrF_2_-10PbO-10M_x_O_y_-0.5Pr6O_11_	10–11	14–17	[15]
M = Li, Na, K, Zn, Bi			
60P_2_O_5_-4B_2_O_3_-7Al_2_O_3_-10K_2_O-17.9BaO-0.1Pr_2_O_3_	-	137	[16]
55SiO_2_-8B_2_O_3_-5Al_2_O_3_-14Li_2_O-2Na_2_O-10GeO_2_-5.9Y_2_O_3_-0.1Pr_2_O_3_	-	73	[16]
75TeO_2_-20ZnO-5Na_2_O-0.1Pr_2_O_3_	-	51	[16]
69H_3_BO_3_-20Li_2_CO_3_-10LiF-1Pr_2_O_3_	25.1	30	[17]
60PbO-40GeO_2_-0.05Pr_2_O_3_	6	145	[23]
5ZnO-15PbO-20WO_3_-59TeO_2_-1Pr_6_O_11_	4.5	-	[27]
44P_2_O_5_-17K_2_O-9Al_2_O_3_-23.9PbO-6Na_2_O-0.1Pr_6_O_11_	-	66	[28]
30PbO-5Bi_2_O_3_-64SiO_2_-1Pr_2_O_3_	69	-	[31]
25Na_2_O-5LaF_3_-10CaF_2_-10AlF_3_-49.9B_2_O_3_-0.1Pr_6_O_11_	-	51	[59]
30Li_2_CO_3_-20Al_2_O_3_-10B_2_O_3_-39.9SiO_2_-0.1Pr_2_O_3_	85.5	108.2	[61]
60TeO_2_-25ZnO-10BaO-4.5La_2_O_3_-0.5Pr_2_O_3_	21	39	[64]
29.95B_2_O_3_-30TeO_2_-16ZnO-10ZnF_2_-7CaF_2_-7BaF_2_-0.05Pr_2_O_3_	45	76	[65]
10Li_2_O-10PbO-9.95Al_2_O_3_-70B_2_O_3_-0.05Pr_6_O_11_	-	165	[66]

**Table 5:** CIE chromaticity coordinates for the studied inorganic glasses doped with Pr^3+^.

Glass Code		CIE Chromaticity Coordinates
(A)	IZSBGL-Pr	x = 0.380; y = 0.327
(B)	GBFG-Pr	x = 0.433; y = 0.370
(C)	GBG-Pr	x = 0.523; y = 0.353
(D)	PPG-Pr	x = 0.582; y = 0.374
(E)	BBG-Pr	x = 0.622; y = 0.366

**Table 6:** Spectroscopic parameters for Pr^3+^ ions in the studied inorganic glass systems.

Glass Code	Spectroscopic Parameters		
	σ_em_ [10^−20^ cm^2^]	σ_em_ × τ_m_ [10^−26^ cm^2^s]	σ_em_ × FWHM [10^−27^ cm^3^]
IZSBGL-Pr	0.50	154	65
GBFG-Pr	0.98	108	206
GBG-Pr	0.97	107	201
PPG-Pr	1.28	77	165
BBG-Pr	0.37	6	81

## Data Availability

The data presented in this study are available on request from the corresponding author.
